# Variational Embedding Multiscale Sample Entropy: A Tool for Complexity Analysis of Multichannel Systems

**DOI:** 10.3390/e24010026

**Published:** 2021-12-24

**Authors:** Hongjian Xiao, Danilo P. Mandic

**Affiliations:** Department of Electrical and Electronic Engineering, Imperial College London, London SW7 2AZ, UK; hongjian.xiao18@imperial.ac.uk

**Keywords:** variational embedding entropy, sample entropy, multi-channel system, physical signal analysis, complexity science, multivariate data

## Abstract

Entropy-based methods have received considerable attention in the quantification of structural complexity of real-world systems. Among numerous empirical entropy algorithms, conditional entropy-based methods such as sample entropy, which are associated with amplitude distance calculation, are quite intuitive to interpret but require excessive data lengths for meaningful evaluation at large scales. To address this issue, we propose the variational embedding multiscale sample entropy (veMSE) method and conclusively demonstrate its ability to operate robustly, even with several times shorter data than the existing conditional entropy-based methods. The analysis reveals that veMSE also exhibits other desirable properties, such as the robustness to the variation in embedding dimension and noise resilience. For rigor, unlike the existing multivariate methods, the proposed veMSE assigns a different embedding dimension to every data channel, which makes its operation independent of channel permutation. The veMSE is tested on both stimulated and real world signals, and its performance is evaluated against the existing multivariate multiscale sample entropy methods. The proposed veMSE is also shown to exhibit computational advantages over the existing amplitude distance-based entropy methods.

## 1. Introduction

We are witnessing a surge in the investigation of structural complexity of real world signals, as complexity science is now recognized to have the same importance as the properties in the time and frequency domains. Indeed, the structural complexity of a data set is a unique feature that can be utilized as a feature to understand subtle changes in the signal generating mechanism via nonlinear analytical tools or through machine learning [1]. Studies employing structural complexity as a feature have covered a wide spectrum, from fault diagnosis of rotating machines [2,3,4] to the early detection of disease and sickness in humans [5,6,7,8]. It is important to note that bio-signals tend to exhibit high degrees of irregularities and complex dynamical behaviours [9], resulting from interactions between the human body (organisms) and peripheral environment, together with continuous fluctuations in time [10]. The complexity loss theory (CLT) has established the potential relationship between the complexity of physical signals and health of an individual, whereby the higher degree of complexity indicates a healthier condition of the individual [11]. However, new developments have declared that pathology may also manifest itself through an increase in complexity, based on the underlying signal structure; that is, a decrease of self-correlated complexity will also be observed in a healthy body [12].

Although the definition of structural complexity is inconsistent in the literature [13], there are several commonly used methods for the quantification of the “degree of dynamics”, with entropy-based methodologies being the most popular ones. Compared to other methods, the estimation of complexity of nonlinear systems, through the fractal dimension [9], recurrence plots [10], and entropy analyses holds the advantage of simplicity and noise robustness [14]. More importantly, complexity based methods do not suffer from any restrictions related to the probability distribution [15]. The features of the loss of complexity (LOC) manifest themselves through, for example, an increase in randomness, reduction in regularity, breakdown of long-term correlations, multiscale variability, and time irreversibility [13]. To this end, a large number of entropy-based algorithms have been proposed to quantify the different facets of complexity or, more precisely, the degree of complexity based on different definitions.

Among numerous existing entropy algorithms, Shannon entropy (SE) and conditional entropy (CE) are the two fundamental methodologies that quantify the amount of information and rate of information generation, respectively [16]. Typical Shannon Entropy-based methods that have been commonly implemented in practical scenarios are permutation entropy [17] and the recently introduced dispersion entropy [18], while the two early widely used conditional entropy-based algorithms are the approximate entropy (ApEn) [19] and sample entropy (SampEn) [20], proposed, respectively, in 1991 and 2000. A modification of ApEn, the SampEn has been shown to reduce the bias experienced by ApEn by removing self-matching delay vectors; SampEn also exhibits less dependency on the data length, thus giving relatively higher consistency [20]. Both ApEn and SampEn were developed to quantify the randomness and irregularity of a signal generating system. Generally speaking, the lower the value of SampEn, the less complex the system.

However, truly complex signals exhibit varying structures, across multiple time scales, while long-range correlations fail to be observed by single-scale sample entropy analysis. To this end, Costa et al. introduced a ‘coarse-graining’ procedure into the sample entropy methodology, to verify the structural complexity hidden in at high scales, referred to as the multiscale sample entropy (MSE) [21]. Despite its broad use, the down-sampling procedure, given by the coarse-graining process, will include artifact components in high frequencies thus generating biased scaled signals [22]. As an improvement of the multiscale entropy strategy, refined multiscale entropy was prposed which uses a low-pass Butterworth filter to generate scaled signals [22]. Although the drawbacks of the coarse-graining process make it impossible to behave as an optimal filter, its simplicity and fast implementation make it valuable in the development of entropy-based applications. This, in turn, further spurred the development of MSE algorithms, including composite multiscale sample entropy [23] and refined composite multiscale sample entropy [24].

However, due to the ‘coarse-graining’ procedure, the requirement of long data length remains even more pronounced and is hard to satisfy in most practical situations. In 2011, multivariate multiscale sample entropy (MMSE) was introduced, which successfully combines data from multiple channels, to estimate the dynamics of the system more accurately and with shorter data lengths [25]. The key improvement of MMSE is the form of composite delay vector, which involves and reconstructs data segments from multiple channels, whereby the inner correlations among diverse signals are preserved [25]. The introduction of MMSE further spurred the research on practical entropy algorithms. The existing multivariate entropies to date include:

- Multivariate multiscale sample entropy (MMSE) [25], a method which performs joint multivariate analysis of physiological signals associated with multiple channels.
- Multivariate multiscale fuzzy entropy (MMFE) [26], which combines composite delay vectors and fuzzy entropy [27] and exhibits smoother and more stable estimates than MMSE.
- Multivariate multiscale permutation entropy (MMPE) [6], an extension of standard permutation dntropy [17] which inherits the desirable properties of PE, such as fast computation and simple implementation.
- Multivariate multiscale distribution entropy (MMDistEn) [28], a recently introduced entropy method, developed on the basis of Shannon entropy with the inclusion of Euclidean distance, which exhibits high calculation efficiency in the quantification of the randomness of system.
- Multivariate multiscale dispersion entropy (MMDispEn) [29], an extension of standard dispersion entropy [18] which is an improvement of permutation entropy with different mapping techniques, which exhibits a more reliable and robust calculation.
- Variational embedding diversity entropy (veMDE) [30], a method developed on the basis of diversity entropy [2] that combines angular distance and relative probability and exhibits a low computational load, with similar performance to MMPE.

Among the existing multivariate entropy algorithms listed above, the last four methods were built based on Shannon entropy, which gives the average uncertainly of a system, while the first two algorithms are based on conditional entropy, which quantifies the generation rate of new information; both were established based on the amplitude of the original signal. Despite success, the inherent shortcomings of amplitude-based CE-developed entropy calculations still remain a major obstacle towards their more widespread use. Other issues with current CE-based multivariate entropy methods include:

1. The rule of thumb is that the requirement for data length is around $10^{m}$ to $30^{m}$, where *m* refers to the embedding dimension [31] for CE-based methods, such as approximate entropy [19], sample entropy [20], and fuzzy entropy [27]. Hence, the choice of the embedding dimension is limited by the available sample size.
2. The ‘coarse-graining’ process further emphasizes the drawback of limited data size, which causes inaccurate and undefined estimation for high scale analysis.
3. Amplitude-based distance between delay vectors is sensitive to outliers, such as noise and artifacts.
4. Poor quality of any single channel has a large impact on the performance in a multivariate setting.
5. Excessive computational burden is required when implementing multi-channel analyses based on CE-developed and amplitude distance calculations.

Recently, Wang et al. [30] introduced a new way to combine datasets from multiple channels into one entropy estimation algorithm, termed variational embedding multiscale diversity entropy. Nonuniform embedding space strategy has existed for a long time in the computation of complexity from different perspectives, such as the estimation of nonlinear causality by corrected conditional entropy [32,33]. When it comes to the multivariate case, the key question is the optimization of the embedding dimension [34]. There is no general answer to this problem, since the optimal embedding dimension for a multi-channel system cannot be unique in practical scenarios [35]. Porta et al have provided a comparison and stated that model-free approaches are less efficient when applied to nonlinear systems under a high embedding dimension; for higher scales, these also exhibit lower reliability [36], due to the lack of available data [34]. Faes et al further introduced a non-uniform approach to detect the nonlinear Granger causality in a multivariate time series by adopting a step-by-step composition of embedding vectors to reduce the conditional entropy [32]. In the method proposed by Wang et al. [30], a simple and intuitive strategy was applied to give the complexity estimation, in terms of irregularity, by constructing the phase space with different structures to generate unique probability distribution for each channel. Here, inspired by [30], based on sample entropy, a new multivariate entropy method is proposed, and is referred to as the variational embedding multiscale sample entropy (veMSE). This new method offers the following advantages over the existing multivariate multiscale sample entropy (MMSE) algorithm:

1. Complexity estimates at a higher embedding dimension are better defined, even with a limited data size.
2. The requirement for the number of data points is lower than in current sample entropy based methods.
3. Strong noise-robustness is exhibited across all scales.
4. The overall performance of a multivariate estimate is independent on the quality of any single-channel within a multivariate dataset.
5. Less computational time is required, owing to a straightforward and efficient implementation.

The aim of this paper is to propose variational embedding multiscale sample entropy (veMSE), a method which combines the different multi-source fusion methods, multivariate strategy, and variational embedding strategy, applied on sample entropy, as well as to demonstrate the merits of the new proposed veMSE. The remainder of the paper is organized as follows. In Section 2, the proposed veMSE algorithm is outlined, and the key improvement of the variational embedding strategy is discussed in detail. Section 3 demonstrates the operation of veMSE on simulated signals, to give an initial insight, with regards to the choice of parameters. Then, based on the suggested parameter setting in Section 3, Section 4 considers and discusses the properties of veMSE, including noise robustness, directionality, and calculation efficiency. Next, veMSE is applied to real-world signals, such as wind and heart rate variability (in Section 5), and compared with the performance of the univariate MSE and MMSE. Finally, conclusions summarise the work in this article.

## 2. Variational Embedding Multiscale Sample Entropy

The steps of the proposed veMSE is given in Algorithm 1. The key advantages of veMSE are:

- It allows for variations in the setting of the embedding dimension for multi-channel signals, as shown in Table 1, while simultaneously maintaining the information within each channel. Compared to MMSE (see Algorithm A1 in Appendix A), despite the fact that different embedding values can also be achieved for each channel by MMSE, veMSE focuses more on the information across data channels. Indeed, the composite delay vector in MMSE successfully combines the embedding vectors of the individual channels within multichannel signals. However, there is a discrepancy and bias between the similarity among the recombined composite delay vectors and those among embedding vectors of the original datasets. The proposed veMSE estimates the complexity information of signals in multiple channels, without involving the cross-information between data channels.
- Due to the varying embedding dimensions, veMSE employs a weighted contribution of multiple channels; that is, the probability distribution of each channel is diverse, which serves to reinforce chaotic features in measured signals of a multi-channel system.
- Since the probability of occurrences of similar patterns with veMSE is processed multiple times, based on the number of channels, and the summation process is performed before the logarithm operation in the last step, the veMSE is theoretically able to unveil the complexity properties under higher embedding dimension, but with the same data length, as compared with existing algorithms based on sample entropy.

To provide insight into the performance of the proposed veMSE, both synthetic signals and real world datasets are considered in the following sections, while the popular MMSE multichannel entropy algorithm is employed as a benchmark for veMSE, under the same conditions.
**Algorithm 1** Variational Embedding Multiscale Sample EntropyAssume that there are *P* channels measured from a system, where a signal, recorded from the *c*th channel, is denoted by $x_{c}\left(i\right)$ and is of length *N*, where 1≤c≤P,1≤i≤N. Parameters involved in the veMSE algorithm are the tolerance quotient (r), embedding dimension (m), scale factor (τ), and time lag (L). The detailed steps of veMSE are shown below.Coarse graining is firstly applied to the original datasets for all the channels, whereby the scaled time series are calculated as $y^{\left(\tau \right)}\left(j\right)=\frac{1}{\tau }\sum_{i=(j-1)\tau +1}^{j\tau }x\left(i\right)$, $1\leq j\leq N_{t},\;N_{t}=\frac{N}{\tau }$.For each channel, the embedding dimension, *m* is set as a variable. The dimension for the *c*th channel is calculated as m(c)=m+c−1, as listed in Table 1. Therefore, combined with the time delay, *L*, the embedding delay vector of data $y^{\left(\tau \right)}\left(i\right)\;\left(1\leq i\leq N_{t}\right)$ for channel *c* is designated as a template, $Y_{c}^{\left(\tau \right)}\left(i\right)$, and calculated as $Y_{c}^{\left(\tau \right)}\left(i\right)=[y^{\left(\tau \right)}\left(i\right),y^{\left(\tau \right)}\left(i+L\right)\;,\ldots ,\;y^{\left(\tau \right)}(i+$$n_{c}$$)],\;n_{c}=\left(m\left(c\right)-1\right)L$.Compute the Chebyshev distance between templates, $Y_{c}^{\left(\tau \right)}$, where the distance is denoted according to the amplitude of the embedding vector, as $d=max\left\{Y_{c}^{\left(\tau \right)}\left(i+k\right)\right)-Y_{c}^{\left(\tau \right)}\left(j+k\right)\}$, where $0\leq k\leq m\left(c\right)-1,\;1\leq i,j\leq N_{t}-n_{c},\;i\neq j$.For each channel, the number of segments, $Y_{c}^{\left(\tau \right)}\left(j\right)$, within the tolerance level, *r*, of $Y_{c}^{\left(\tau \right)}\left(i\right)$, is recorded as $B_{c}\left(i\right)$. In other words, $B_{c}\left(i\right)$ is the number of templates matched or the number of similar patterns in the dataset, based on the boundary set, where the boundary is defined by the tolerance, *r*. Therefore, the local probability of the occurrence of template match for channel *c* is $R_{c}\left(i\right)=\frac{B_{c}\left(i\right)}{\left(N_{t}-n_{c}-1\right)}$.The global probability of the occurrence of template match for a channel *c* is calculated as $\Phi \left(c\right)=\frac{1}{\left(N_{t}-n_{c}\right)}\sum_{i=1}^{N_{t}-n_{c}}R_{c}\left(i\right)$.For all data channels, compute the sum of the global probability as $\Phi =\sum_{c=1}^{P}\Phi (c)$. Recall that, unlike the MMSE algorithm (see Appendix A), the embedding dimension, m(c), is not a fixed parameter and is varied, along with the index of channel, *c*.Modify the embedding dimension to (m(c)+1). Hence, $n_{c}$ is adjusted to m(c)×L, and Steps 2–6 are repeated to obtain the global probability for the so-increased embedding dimension Φ(m+1).Variational embedding multiscale sample entropy is finally obtained, as $veMSE=-ln\frac{\Phi (m+1)}{\Phi (m)}$.

## 3. Results of veMSE on Stimulated Signals

In this section, synthetic signals, generated based on five benchmark models, are utilized to illustrate the performance of veMSE. These include white Gaussian noise (WGN), flicker noise (coloured noise), and autoregressive (AR) models AR(1), AR(2), and AR(3). Standard deviations for all the generated signals were set to σ=1, while the coefficients of AR models are given in Table 2. The role of the parameters discussed in the following subsections include the embedding dimension, data length, tolerance, number of channel, and scale factor. Essentially, the temporal span is jointly controlled by the embedding dimension and time delay. Here, to avoid unknown influence of control variables, time lags were set to L=1 for all operations, to make the temporal span fully defined by the modification of the embedding dimension.

Figures in each subsection are presented in pairs, to illustrate the structural complexity results for the five benchmark models, and compared with the standard MMSE algorithm. Upper panels give the curves for white and flicker noise, while the panels in the bottom depict the results of the AR models, in contrast to white noise. Complexity curves of entropy values are plotted as error bars, based on standard deviation, averaged over outcomes of 20 independent realizations for each model.

### 3.1. Varied Embedding Dimension (m)

**Figure 1:**
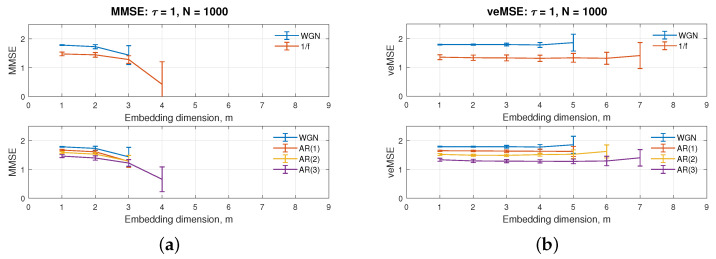
Behaviour of the single-scale (**a**) MMSE and (**b**) veMSE, on the estimation of WGN, 1/f noise, and AR processes, as a function of the embedding dimension, m.

Usually, for the implementation of SampEn-based algorithms, the embedding dimension and data length are the two parameters that are interdependent and mutually coupled, with the data size restricted to between $\left(10^{m}-30^{m}\right)$, as a rule of thumb [37]. In the real world, the recorded signals do not have infinite length and are generally limited by operation time and memory space. Therefore, the embedding dimension is commonly set to quite low values of m=2 or m=3 for a signal with 1000 samples [38]. Higher values of the embedding dimension for a small data size will cause unstable estimation, as in standard MMSE, shown in Figure 1a.

The corresponding results for the veMSE, as a function of embedding dimension, are shown in Figure 1b. Each entropy estimate was calculated based on signals from two channels. Except the independent variable, *m*, other parameters, such as the scale factor and data length, were set as constant values (1 and 1000, respectively). The tolerance, *r*, was varying, according to the total variance of the covariance matrix of processed data sets, as r×tr(S); here, the tolerance quotient was fixed to r=0.15.

Figure 1b, where the embedding dimension ranges from 1 to 9, shows that, unlike MMSE, the veMSE analysis was able to give a defined entropy value, even at high embedding dimensions and for a complex correlated structure, as, e.g., the AR(3) process at the scale of 7. Additionally, with standard MMSE, signals with higher randomness are more likely to yield unstable estimates as the embedding dimension increases. However, even in the case of white Gaussian noise with the highest randomness, the veMSE with the embedding dimension *m* = 5 was able to successfully and stably process the data. On the other hand, traditional multiscale sample entropy methods fail to give a defined value with the embedding dimension higher than 3, under the same condition [12]. Therefore, from the viewpoint of estimation stability, veMSE exhibits a marked improvement, when it comes to assessing complex information in high dimensions.

### 3.2. Varied Data Length (N)

The data length, *N*, of the signal is another limitation, in addition to the embedding dimension, when implementing entropy-based complexity calculations, particularly in real world processes. Indeed, amplitude distance-based entropy algorithms require at least 1000 data points to guarantee a consistent estimation, such as with the multiscale sample entropy (MSE) and multiscale fuzzy entropy (MFE) [27]. However, in real world data, as in the analysis of heart rate variability, for example, to obtain the required data size for RR intervals, a minimum of 5 min of the raw electrocardiograph (ECG) signals are needed. In practice, the implementation of such a long-time recording in a controlled state is hard to be satisfied. Compared to amplitude distance-based entropy methods, space distance-based entropy algorithms, such as cosine similarity entropy, show less restriction to data length, with a minimum of 700 samples required [12].

**Figure 2:**
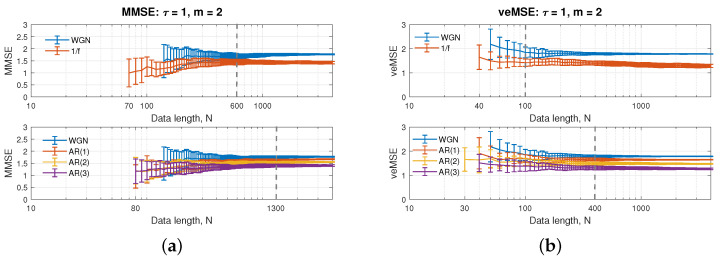
Behaviour of the single-scale (**a**) MMSE and (**b**) veMSE, on the estimation of WGN, 1/f noise, and AR processes, as a function of the data length, *N*.

Figure 2b illustrates the performance of a single scale of veMSE, as a function of data length, *N*, in a logarithmic scale. The embedding dimension was set to m=2, and the choice of tolerance was the same as before. The values of veMSE for white and 1/f noise were not defined before *N* = 40, while for AR(2), they were not defined when *N* was smaller than 30. The smallest sample sizes for the veMSE to reliably compute entropy estimates for AR(1) and AR(3) were also *N* = 40.

Observe that the standard deviation of the entropy results is gradually decreasing with an increase in data length, while the range for each error bar is increasing from WGN to AR(3). As a result, a system with more structure (AR(3)) reveals a larger standard deviation. In addition, the consistency of the estimation can be guaranteed as evidenced by the relative position of curves in each graph being unchanged as data length, *N*, increases. More importantly, when analysing the white and flicker noise, in the top panel in Figure 2b, the estimation at the *N* = 100 sample length could successfully separate the complexity degrees of the two signals, while with veMSE, in the bottom panel in Figure 2b, when the data length reaches *N* = 400 samples, there is no intersection region among entropy values from different models. This illustrates that the requirement of data length when applying veMSE is much lower than in other entropy methods, e.g., MMSE in Figure 2a requires *N* = 1300 for the separation of the four models. Subsequently, this property enables us to reveal the complexity information under high scales, even at a limited data length, but with a stable estimation, which better serves the balance between the dimension and data size.

### 3.3. Varied Tolerance (r)

The tolerance, *r*, can be explained as the boundary of the similarity degrees among comparing templates. The SampEn-based algorithms limit the tolerance to a hard threshold as a Heaviside function related to the standard deviation of the original data. However, for a multivariate case with multichannel data sets, only a single tolerance value is allowed in standard algorithms. As in [25], the choice of tolerance of veMSE is dependent on the total variance of the covariance matrix, ***S***, of the analysed data sets. Therefore, the tolerance was set as r×tr(S).

**Figure 3:**
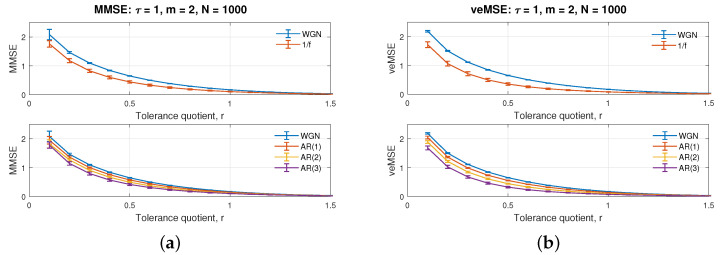
Behaviour of the single-scale (**a**) MMSE and (**b**) veMSE, on the estimation of WGN, 1/f noise, and AR processes, as a function of the tolerance, *r*.

Figure 3b illustrates single-scale entropy estimation, as a function of the tolerance parameter, *r*, varying from 0.1 to 1.5, at 0.1 increments. The data length and embedding dimension were fixed at *N* = 1000 and *m* = 2, to show the influence of the varied tolerance setting. Observe from the figures that, for all curves, the increase of tolerance quotient results in a monotonic decrease in complexity estimation, which is the same behaviour as with MMSE, in Figure 3a. All curves can be obviously distinguished from each other, before *r* = 1 in veMSE, with the values after *r* = 1 too small for differentiation. The gap among different complexity estimations in the two figures obviously narrows down after *r* = 0.5, therefore, supporting the choice for the value of tolerance quotient to be chosen below *r* = 0.5.

### 3.4. Varied Number of Channels (P)

**Figure 4:**
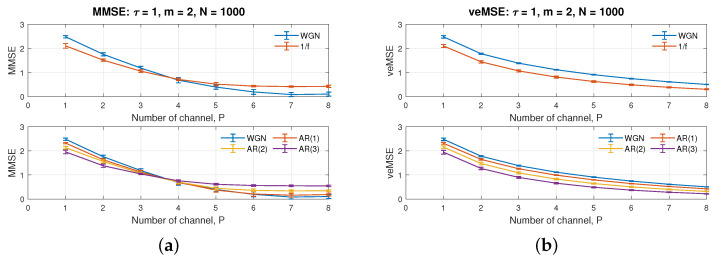
Behaviour of the single-scale (**a**) MMSE and (**b**) veMSE, on the estimation of WGN, 1/f noise, and AR processes, as a function of the channel number, *P*.

The number of channels, *P*, is closely related to the setting of the embedding dimension, *m*, and the performance of veMSE. We have showed that the veMSE is able to give complexity estimation under higher embedding dimension in Figure 1b, the higher embedding dimension will be assigned to consecutive data sub-channels. Figure 4a,b illustrate the influence of the number of data channels on MMSE and veMSE. The default parameters were set to *m* = 2, *N* = 1000, and τ = 1.

Figure 4 illustrates single-scale estimation, as a function of the channel number, *P*, varying from 1 to 8. Observe that all the curves exhibit a decreasing trend for both algorithms, yet for different reasons. The drop in MMSE is resulted from the low level of correlation within the signals. Hence, the increase of channel number in MMSE has the same effect as the increase of scale factor, exhibited in the next sub-section. The decrease in veMSE is caused by the absence of information, hidden in high embedding dimensions, and simplicity of the system structure; that is, the extra channels fail to give more information, which could contribute to the complexity estimation. Despite this decrease, the estimation of veMSE is consistent for all the situations. The gap among different signals in Figure 4 keeps at a certain level for a high number of channels. Although the increasing channel number has limited impact on the ability of veMSE to separate systems, with various degrees of regularity, a too high embedding dimension, given by the large number of channel, might possibly fail to provide new information, due to no similar patterns in the following sub-channels. Therefore, according to the discussion in Section 3.1, the ideal uncorrelated WGN holds a well-defined value for embedding dimension, *m* = 5. We suggest the number of channels, ranging from 1 to 5, for the best performance of veMSE. In short, veMSE is more suitable for analysis of systems with limited number of channels and strongly correlated inter-channel structure.

### 3.5. Varied Scale Factor

As noted by Costa et al. in [21], the multiscale analysis by integrating consecutive coarse-graining is of importance in signal processing associated with hidden correlation structure in data. Based on the aforementioned analysis of parameters involved in veMSE, the embedding dimension was set to *m* = 2, and the tolerance was chosen as *r* = 0.15, multiplied by the total variance of the covariance matrix. With regard to performance of multiscale analysis, graphs of multichannel entropy results are presented, in response to the scale factor, varying from τ = 1 to τ = 40. Dual channel (bivariate) data, with *N* = 3000 data points for each model, were considered.

**Figure 5:**
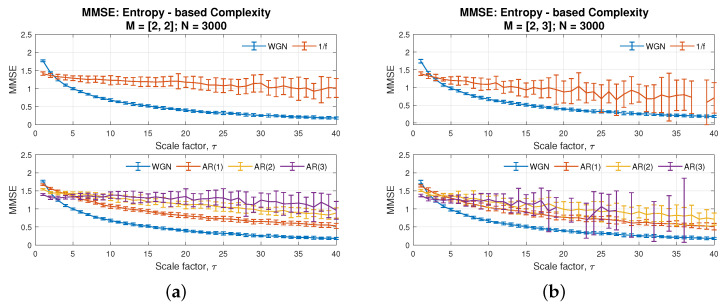
Behaviour of Multivariate Multiscale Sample Entropy [25], for WGN, 1/f noise, and AR processes, with the embedding dimension set as (**a**) [2, 2] and (**b**) [2, 3].

**Figure 6:**
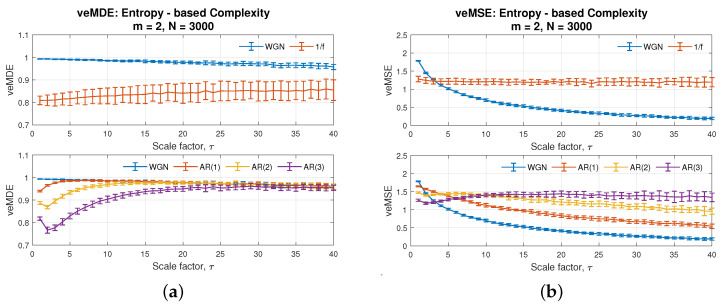
Behaviour of (**a**) variational embedding multiscale diversity entropy (veMDE) [30], and (**b**) the proposed variational embedding multiscale sample entropy (veMSE), for WGN, 1/f noise, and AR processes, with the embedding dimension set as *m* = 2.

To further elucidate the extent of improvements of the proposed veMSE, over the multivariate multiscale sample entropy (MMSE), its performances were compared against the proposed veMSE method. With the same data size, due to the varying embedding dimension feature of veMSE, two different settings of the parameter, related to embedding dimension for MMSE, were applied and are shown in Figure 5a,b. In addition, the performance of the variational embedding multiscale diversity entropy (veMDE) is also given in Figure 6a, with the embedding dimension set to *m* = 2, while the results of veMSE are presented in Figure 6b.

These figures demonstrate that complex structure, hidden in higher dimensions, is hard to unveiled via MMSE, as the standard deviation of those independent 20 realizations grows steadily as the scale factor increases. Overall, the embedding dimension pair [2,2] gives a better performance in MMSE for the considered restricted data length. However, even under the optimal dimension settings, as in Figure 5a, the complexity of AR(3) in purple and AR(2) in yellow fails to be distinguished in multi-scale cases. As for the veMDE in Figure 6a, diversity dntropy is developed based on angular distance and Shannon entropy, which measures a complex system from a different perspective (by the amount of information [2]). As can be seen in the graph, veMDE reveals a consistent estimation for each system considered, which exhibit short-term correlation. Our analysis focuses on the improvements of variational embedding fusion methodology over multivariate multiscale strategy, based on sample entropy. Details of the veMDE improvements by the variational embedding strategy, compared to other existing entropy methods, can be found in [30].

On the other hand, the merits of veMSE can be clearly seen from Figure 6b. To better specify the improvement, the optimal dimension setting [2,2] of MMSE in Figure 5a was utilized for a comparison with veMSE. Observe from the top panel of the two types of noise in each figure. Although both of the two algorithms were able to distinguish between the two models, the complexity of white noise went down, while the flicker noise maintained a certain complexity level, in spite of the increasing scale. The range of entropy in error bars for flicker noise based on veMSE was much narrower than that in basis of MMSE, especially in large scales. Secondly, in the bottom panel in each figure, values of AR(2) and AR(3) (in yellow and purple) fail to be fully separated by MMSE in the cases of high scale, as stated above, while with the same data length, the separability of AR models of different orders was successfully accomplished in the proposed veMSE. It is critical to apply the entropy calculation under the multiscale situation, since the long-range correlation of the system is largely ignored in the analysis under low scale. Next, it can be observed that minor differences exist between the complexity estimation of the two models, namely white noise and AR(1) (blue and red line in bottom graph). Instead, the enhancement properties of veMSE is particularly revealed in the analysis of highly correlated and structured signals, as well as systems with higher structural complexity.

Overall, the comparison of veMSE and MMSE, based on the above five models, shows that the veMSE provides a more stable estimation that can better demonstrate complex temporal fluctuations. In addition, veMSE is especially suitable for multiscale analysis of highly correlated signals, which exhibit variation of spatial–temporal patterns over a range of scales.

## 4. Properties of veMSE

**Figure 7:**
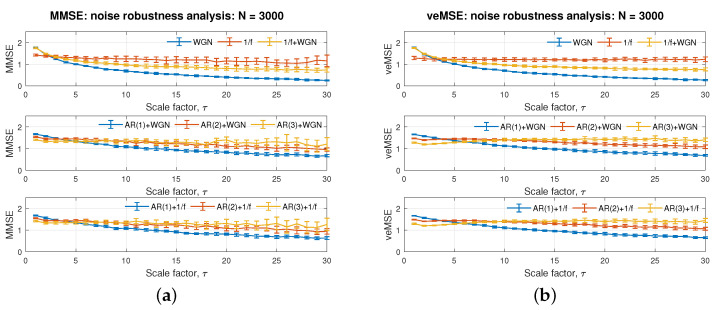
Illustration of robustness, with respect to noise on (**a**) standard MMSE and (**b**) the proposed veMSE.

**Figure 8:**
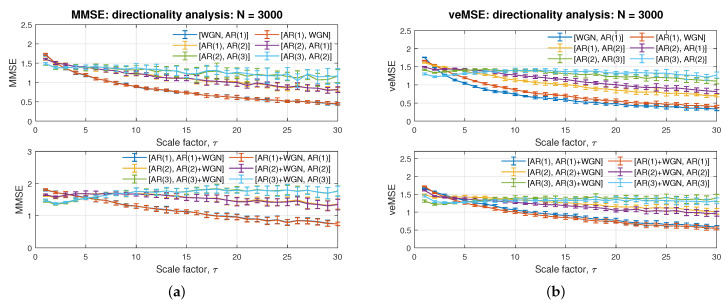
Influence of directionality on (**a**) standard MMSE and (**b**) the proposed veMSE.

**Figure 9:**
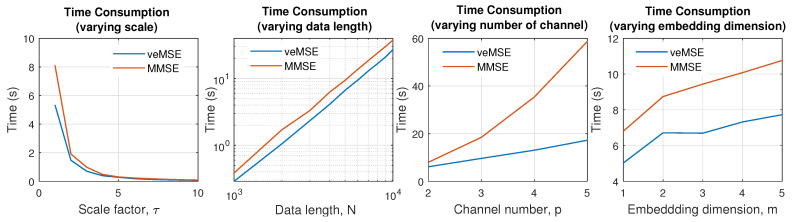
Computation time for MMSE and the proposed veMSE, with the modified parameters for a white Gaussian input.

We now elaborate on the three desired properties of the proposed entropy based veMSE algorithm: noise robustness, directionality, and calculation efficiency. The parameters setting was as follows: data size *N* = 3000; embedding dimension *m* = 2; tolerance *r* = 0.15; and scale factor τ = 1. A bivariate system was considered in the analysis. The results which depict the noise analysis and directionality analysis based on the proposed veMSE are presented in Figure 7b and Figure 8b, respectively. The corresponding performance of MMSE is shown in Figure 7a and Figure 8a. The time requirement for the calculations of veMSE and MMSE is shown in Figure 9.

### 4.1. Noise Robustness

Robustness against noise and artifacts is of critical importance in any estimation. Given that it is infeasible to avoid the noise associated with recording equipment and the ubiquity of artifacts in biosignals, for instance, muscle and electro-magnetic artifacts exist in EEG-based monitoring [39], the noise-robustness property was tested, by comparing the complexity estimation for AR models with and without corrupting noise. In Figure 7b, the top panel presents the curves for uncorrelated white Gaussian noise (WGN), correlated flicker noise (1/f noise), and coloured noise, containing both WGN and 1/f noise. Observe that the three systems with different degrees of correlation were successfully separated by the veMSE. Adding white noise will enhence short-term correlation, as shown at the left of the top panel in Figure 7b, where the yellow line (1/f + WGN) is as high as the blue line (WGN), while the long-term correlation is lower as the scale factor increases (1/f>1/f+WGN> WGN). The top panel in Figure 7b reveals that veMSE could correctly yield complexity estimation, in line with the theoretical analysis, on the basis of uncorrelated and correlated noise.

In the middle and bottom panel in Figure 7b, the results of veMSE for AR models with uncorrelated white noise and correlated flicker noise are presented, to contrast to the outcomes of pure AR signals in Figure 6b. The amplitude of the added noise signal was set to 20% of that for the AR signals. Compared to Figure 6b, the gaps between the complexity curves for the AR models of varying order decrease with noise. However, even that the gap among distinct models is narrowed down, separation can still be achieved at high scales in 20% of the noisy scenarios, while in case of MMSE, noisy AR signals with different complexity cannot be well separated and the impact of noise is clearly shown in Figure 7a. Given these points, the performance of complexity estimation, based on veMSE, is consistent with cases without noise, a unique feature of veMSE that is not present in the other MSE algorithms, thus demonstrating the potential in practical recording data sets.

### 4.2. Directionality

For multivariate analysis, the directionality refers to the issue that the optimal ordering of the input channels is unknown; it is, therefore, desirable that an algorithm is independent of channel ordering. Yet, without prior knowledge related to the optimal channel order, the performance of the estimation will be impacted in standard entropy-based algorithms. To this end, the directionality of the veMSE is next analysed for bivariate systems. Figure 8b shows two graphs, each containing three pairs of curves. The top panel depicts the results for white noise with AR(1), AR(1) with AR(2), and AR(2) with AR(3), with the order of input shown by legend (first present, first processed). As can be seen from this figure, the estimates at a lower scale are mainly influenced by the first input signal. For example, the blue line [WGN, AR(1)] can be clearly recognized as lower than the red one [AR(1), WGN] at the beginning, especially in the single-scale case. As the scale increases, the two lines approach the same level; a similar trend can be seen for the other two pairs.

In the bottom panel in Figure 8b, the analysed signals are AR(1), AR(2), and AR(3), with one of the signals in each system associated with white noise. The legend [AR, AR+WGN] refers to cases where noise-free signals are the first variate, followed by noisy signals, and vice versa for [AR+WGN, AR]. This setting of the inputs was used to simulate real world scenarios when dealing with multi-channel signals, whereby one of the constituent channel represents a poor recording with noise. Considering the noise-robustness property of veMSE, the amplitude of noise signals in this subsection was enlarged to the same level as for the AR signals, to demonstrate a clear difference when the input order is altered. As shown in the figure, the inverted input orders can be reflected by different start levels, while the complexity curves then approach each other, as well as ending with similar estimates. Therefore, regardless of the input order, the separation of complexity levels of AR models was successfully achieved with the proposed veMSE algorithm, as observed in Figure 8a. In the case of MMSE, the inverted input exhibited no influence on the resulted curves in small scales, while similar performance as veMSE in the larger scale.

As demonstrated in Figure 8b, for the proposed veMSE, the reversed order had little influence on the estimation at high scales as all the paired curves approach to the same three regions, so that, in spite of the modified order, the three models were separated. However, a similar phenomenon is shown in the top panel in Figure 8b, the varying order of the input signals will generate entropy values with different degrees at small scale analysis when the input signals contain distinct structure. Therefore, the direction of the input order needs to be carefully considered when applying small scale analysis, and such considerations can be ignored at high scale analysis with identical system measurements.

### 4.3. Computational Complexity

Entropy analysis based on multichannel signals is more time-consuming than single variate estimation, so that calculation efficiency becomes one of the critical factors that needs to be carefully considered. Therefore, in this subsection, time consumption of veMSE is discussed and compared with the commonly used MMSE.

Figure 9 shows the processing time, as a function of various modified parameters, when implementing the veMSE (blue line) and MMSE (red line). All the curves are produced as an average over 10 independent realizations. Each graph is designed to reflect the behaviours for only one modified parameter, with the independent variables in the following figures, from the left- to the right-hand side, as the scale factor, length of data (in log-log scale), number of channels, and embedding dimension. All the entropy calculations were set as single-scale and bivariate processing by default. The data length and embedding dimension were irrelevant variables and were fixed to *N* = 5000 and *m* = 2.

Overall, the red line, representing the computational time of the standard MMSE, was above the blue, that of veMSE, for all the scenarios. The increase of scale factor reflects that when the data size, after ‘coarse-graining’ procedure, is lower than *N* = 1000, the times needed for the two calculations are similar, as shown in the left most graph where the scale factor is higher than 5. The relation between the computational load and data length in the second graph from the left indicates that veMSE has the dependency of $O(N^{2})$ on the data size as standard MMSE, given in the log-log scale plot. As for the influence of the number of channels, demonstrated in the third graph from the left, it is reasonable that MMSE needs more time as the channel number increases, because the key step for sample entropy is the ratio of conditional probability for similar patterns between the embedding dimension, *m*, and its increment, m+1, whereby the number of possible ways to apply the (m+1)-dimension is equal to the number of channels involved when forming the composite delay vector in MMSE. Therefore, the calculation, with an increased embedding dimension, will be repeated *c* times in MMSE, where *c* denotes the number of data channels. Finally, in the relationship between an increased embedding dimension and computation time in the right-most panel, the time difference roughly maintains a fixed value, in spite of the embedding dimension changing.

In summary, compared to the widely used MMSE, the time needed for the same amount of data with the proposed veMSE is shorter. Therefore, the calculations efficiency of veMSE is higher than that of MMSE, which gives it high potential in real-time monitoring of human states.

## 5. Performance of veMSE on Real Signals

### 5.1. Wind Dynamics

Having illustrated the advantages of veMSE on synthetic signals, we shall now examine the performance of veMSE in real-world systems. We first considered three-variate wind data of different dynamics. The long-term correlations in wind dynamics has been revealed in previous studies by using detrended fluctuation analysis (DFA) [40] and standard multivariate multiscale sample entropy (MMSE) [25].

Here, the proposed veMSE showed an improved performance in characterizing different dynamical complexity of wind regimes.

**Figure 10:**
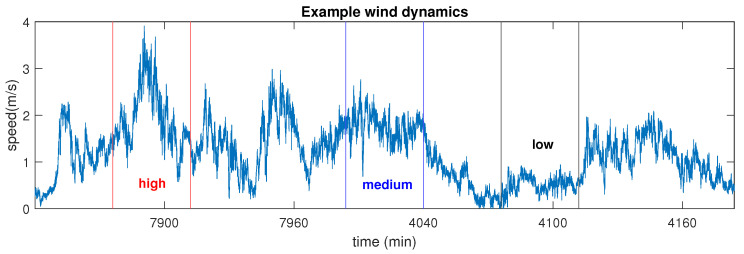
Magnitude of the wind signal considered. The wind segments are defined as the low, medium, and high regimes.

Wind data was recorded by 3D ultrasonic anemometers, at a sampling frequency of 50 Hz. The recording process was implemented in the Institute of Industrial Science of the University of Tokyo [25]. Three channels, containing wind speed into the east-west, north-south, and vertical direction, were recorded. The wind regimes, containing different dynamics, were defined as the low, medium, and high by the magnitude of wind speed, as shown in Figure 10. The parameters involved in veMSE analysis were set to *m* = 2, *L* = 1, r=0.2×tr(S), and *N* = 3000. Illustrations of entropy results were averaged over 10 trials for each channel and displayed as error bars, based on the standard error of the mean. In addition, time-shuffled wind data sets were also tested and compared with the obtained wind dynamics. The shuffled wind samples were generated from the recorded wind signals but with a randomised order. In this case, the possible correlations within signals were broken down but the statistical properties were preserved.

**Figure 11:**
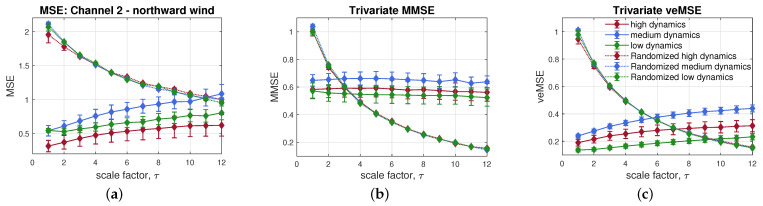
Complexity analyses of real-world wind data; (**a**) Standard single-variate MSE; (**b**) Standard multi-channel MMSE; (**c**) Proposed multivariate veMSE.

Before performing the comparison between veMSE and MMSE, univariate multiscale sample entropy (MSE) was firstly applied to give an insight into the complexity of the dynamical system to be analyzed. As shown in Figure 11a, the randomized data sets exhibit a white noise-like behaviour, in terms of structural complexity, which is expected, as the correlation was destroyed while shuffling data samples. The MSE results of wind dynamics assigned the highest complexity to the medium dynamics regime, which is expected, since the medium wind speed held the fewest constrains. The complexity of low wind regime was quantified as higher than that of the high regime, which is against the intuition, as the high wind regime also contains components with medium speed. Besides, each wind dynamics exhibited lower complexity than the their randomized series, wrongly suggesting the absence of long-term correlations within the wind data sets.

The same trails were analyzed by multivariate multiscale sample entropy, as shown in Figure 11b, which revealed the relation of the three wind regimes in an expected way; that is, with mild wind exhibiting the highest complexity, followed by the high and low dynamics regimes. Further, all the three wind regimes were able to present higher long-range correlation than their surrogate data sets, after τ = 5; however, the overlapped areas can be found across all the scales. Observe from Figure 11c that, as desired, although the scale factor need to be set up to 11, the long-range correlation of different wind speeds was detectable by the proposed veMSE, resulting in a more obvious separation among the different wind regimes, as compared to MSE and MMSE.

### 5.2. Physiological Data

The physiological signals utilized were from the Fantasia database [41], which includes the RR intervals (RRI) and interbreath intervals (IBI), extracted from the electrocardiogram (ECG) and respiration signals, respectively. The structure of long-term correlation in heart rate variability (HRV) and respiratory dynamics was examined by traditional methods, namely detrended fluctuation analysis (DFA) [42] and standard MMSE [25].

The Fantasia database contains recordings from 20 young people (the age of 21–34) and 20 elderly participants (ageing from 68–85) who were rigorously screened for 120 min; we chose 10 subsets from each group. The ECG and respiration signals were recorded at the sample frequency of 250 Hz. Then, RRI and IBI signals were extracted and aligned, and the surrogate series with randomised order were analyzed, along with the RRI and IBI signals for each methodology, to reveal the effectiveness of the complexity analysis. The parameters in entropy analysis were set to *m* = 2, *L* = 1, r=0.15×tr(S), and *N* = 4000.

**Figure 12:**
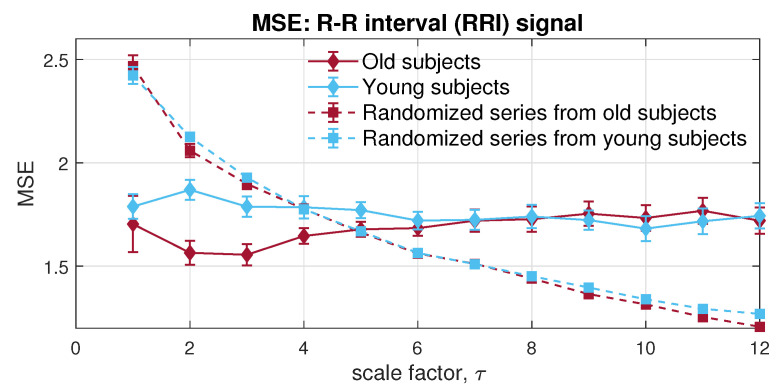
Behaviour of univariate multiscale sample entropy, applied to RR intervals within the Fantasia database.

**Figure 13:**
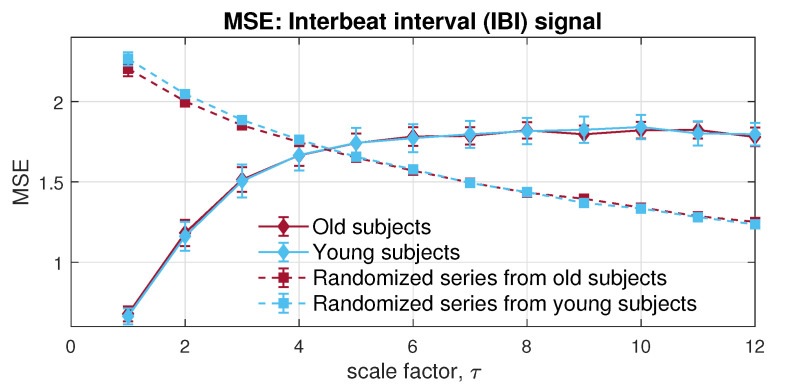
Behaviour of univariate multiscale sample entropy for the interbreath intervals (IBI) within the Fantasia database.

In the first place, to give an overall view of the signals in each channel, univariate multiscale sample entropy was applied to single channels of RRI and IBI, separately, as shown in Figure 12 and Figure 13. As complexity loss theory [43] states, the adaptive capacity of bio-systems is damaged by disease and aging. The complexity of the RRI and IBI signals recorded from young people is, therefore, supposed to be higher than that from elderly subjects, when considering long-term correlation. Regarding short-term correlation in RRI, the MSE exhibited correct estimation under certain low scales. However, the MSE for RRI and IBI was unable to reveal the correct relation between the dynamics of elderly individuals and young people in long-range terms.

**Figure 14:**
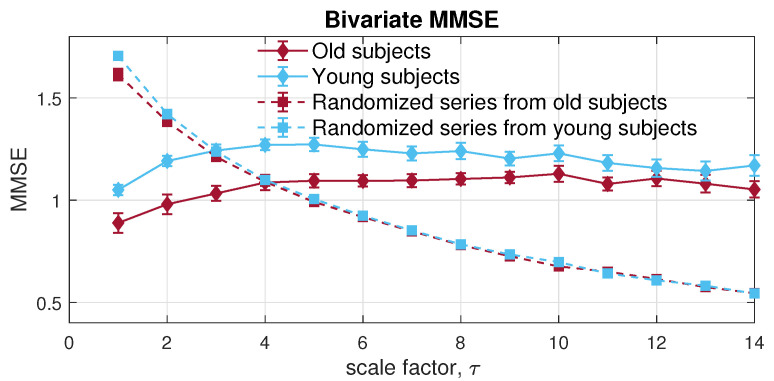
Behaviour of multivariate multiscale sample entropy for the bivariate [RRI, IBI] data from the Fantasia database.

**Figure 15:**
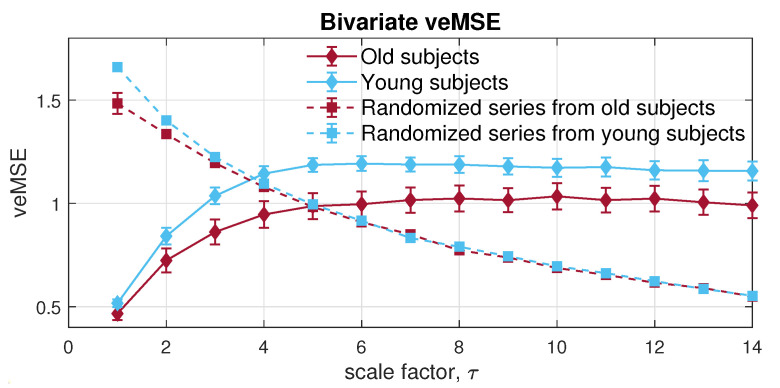
Behaviour of the proposed variational embedding multiscale sample entropy for the bivariate [RRI, IBI] data from the Fantasia database.

Next, the MMSE was applied to the bivariate channels composed of the RRI and IBI signals. Observe from Figure 14, the resulted error bars indicate higher dynamical complexity in young participants than of that in elderly individuals, which conforms with the complexity loss theory due to aging [43]. Moreover, both physiological data sets showed higher long-range correlation than the randomized surrogate series in an expected way; yet, as scale increases, an overlapping area can be observed, due to the lack of sufficient sample length at larger scales, owing to the coarse graining process. In contrast with MMSE, the veMSE in Figure 15 gave the correct and clear estimation, with enhanced stability, particularly in large scales. In practical scenarios, the size of recorded signals is mostly limited; hence, the veMSE better facilitates the analysis of physical and physiological signals when identifying dynamical differences based on nonlinear features.

## 6. Conclusions

The variational embedding multiscale sample entropy (veMSE) method has been introduced for robust structural complexity analysis of real-world data. It has been shown that veMSE is capable of assessing the complex features of the system at large scales and with higher embedding dimensions, compared to the standard MMSE. In addition, the utilization of multivariate analysis via veMSE guarantees an improvement over single-variate analysis, regardless of the quality of the recorded signals in sub-channels. The veMSE has also been shown to exhibit strong noise robustness and lower computational complexity than MMSE, under the same conditions. As desired, this improvement is apparent as the number of available channel increases within a certain range. The higher calculation efficiency within veMSE is of high interest when applying entropy analysis in scenarios which require near real-time processing or synchronized monitoring.

However, a very large number of channels will lead to inefficient measures, while the common problem of the sample entropy-based method still remains, that is, the irregularity of the signal is insufficient to quantify the complexity of the system. Therefore, future work can be considered from several aspects. First, the scaling process, applied in this method, will be further examined, while the multi-scale procedure can be further improved by balancing on computational efficiency. Secondly, the choice of time lag needs to be further explored, to contribute to optimal temporal span. Thirdly, as already stated, to further explore the property of complexity, the estimation needs to be considered from various perspectives, other than the current irregularity-based analysis, such as causality, determinism, nonlinearity, and other features. Lastly, it should be noted that the veMSE method is restricted to amplitude-based distance. Future research will focus on angular distance-based algorithms, which employ the variational embedding dimension methodology.

## Figures and Tables

**Table 1:** Relation between the embedding dimension (*m*) and index of channel (*c*).

Index of channel (c)	1	2	…	*c*
Embedding dimension (m(c))	*m*	m+1	…	m+c+1

**Table 2:** Coefficients of the AR models used.

Coefficients	$a_{1}$	$a_{2}$	$a_{3}$
AR (1)	0.5	−	−
AR (2)	0.5	0.25	−
AR (3)	0.5	0.25	0.125

## Data Availability

The data that support the findings of this study are openly available in figshare at https://doi.org/10.6084/m9.figshare.17429693.v1 (accessed on 24 October 2021).

## Appendix A. Algorithm of Multivariate Multiscale Sample Entropy

**Algorithm A1** Multivariate Multiscale Sample Entropy
The steps of standard Multivariate Multiscale Sample Entropy are given below, for a multi-variate data set ${\left\{x_{c,i}\right\}}_{i=1}^{N},\;1\leq c\leq P$ of length *N* and number of channel *P*. The manually selected parameters are the embedding dimension $M=[m_{1},m_{2},\ldots ,m_{P}]$, tolerance *r*, time delay $L=[l_{1},l_{2},\ldots ,l_{P}]$, and scale factor τ: 1. Normalize the original multi-variate data sets by subtracting the mean and dividing by the standard deviation. 2. Perform Coarse Graining Process to obtain the scaled multi-channel time series ${\left\{y_{c,i}^{\left(\tau \right)}\right\}}_{j=1}^{N/\tau }$, according to $y_{c,i}^{\left(\tau \right)}\left(j\right)=\frac{1}{\tau }\sum_{i=j-\tau /2-1}^{j+\tau /2-1}x_{c}\left(i\right),\;1\leq j\leq \frac{N}{\tau },\;c=1,2,\ldots ,P$. 3. Form the Composite Delay Vectors (CDV) $Y_{M}\left(i\right)$ according to *M* and *L* in the form $$\begin{array}{cc} Y_{M}\left(i\right)= & [y_{1,i},y_{1,i+l_{1}},\ldots ,y_{1,i+\left(m_{1}-1\right)l_{1})},\;\\ & y_{2,i},y_{2,i+l_{2}},\ldots ,y_{2,i+\left(m_{2}-1\right)l_{2})},\;\\ & \;\;\;\vdots \;\\ & y_{c,i},y_{c,i+l_{c}},\ldots ,y_{c,i+\left(m_{c}-1\right)l_{c})},] \end{array}$$ 4. Compute the similarity for all pairwise CDVs, $Y_{M}\left(i\right)\;\&\;Y_{M}\left(j\right)$, based on the Chebyshev distance as $D\left(i,j\right)=max\{Y_{M}\left(i+k\right)-Y_{M}\left(j+k\right)||\;0\leq k\leq \left(\left(\sum_{c=1}^{P}m_{c}\right)-1\right),i\neq j\}$. 5. Calculate the number of matching patterns, defined as similar pairs B(i) that satisfy the criterion D(i,j)≤r. 6. Compute the local probability, C(i), and global probability, Φ(i), of B(i) as $C\left(i\right)=\frac{B(i)}{N-n-1},\;\Phi =\frac{\sum_{i=1}^{N-n}C(i)}{N-n},\;n=max\left(M\right)*max\left(L\right)$. 7. Repeat Steps 3–6 with an increased embedding dimension, $m_{c}$+1, and obtain the updated global probability as $\Phi^{*}=\frac{\sum_{i=1}^{N-n}C^{*}\left(i\right)}{N-n},\;n=max\left(M^{*}\right)*max\left(L\right)$. Recall that there are *P* ways to increase the embedding dimension and the modified global probability, $\Phi^{*}$, is the averaged result. 8. Multivariate Multiscale Sample Entropy is defined as $MMSE=-\ln\left[\frac{\Phi^{*}}{\Phi }\right]$.
